# Detection of *Clonorchis sinensis* Circulating Antigen in Sera from Chinese Patients by Immunomagnetic Bead ELISA Based on IgY

**DOI:** 10.1371/journal.pone.0113208

**Published:** 2014-12-04

**Authors:** Ge Nie, Ting Wang, Shengjun Lu, Wenqi Liu, Yonglong Li, Jiahui Lei

**Affiliations:** Department of Parasitology, School of Basic Medicine, Tongji Medical College, Huazhong University of Science and Technology, Wuhan, China; University of Illinois at Chicago, United States of America

## Abstract

**Background:**

Clonorchiasis, caused by *Clonorchis sinensis*, is widely distributed in Southeast Asia including China. Clonorchiasis is included in control programs of neglected tropical diseases by World Health Organization (WHO) because it is one of the major health problems in most endemic areas. Diagnosis of clonorchiasis plays a key role in the control programs. However, so far, there is no satisfactory method for clonorchiasis because of low sensitivity, poor practicality and high false positivity of available diagnostic tools.

**Methodology/Principal Findings:**

We developed an immunomagnetic bead enzyme-linked immunosorbent assay (ELISA) based on IgY (egg yolk immunoglobulin) against cysteine proteinase of *C. sinensis* for detection of circulating antigen in serum samples of patients infected with *C. sinensis*. The polyclonal IgY, coated with magnetic beads, was used as a capture antibody and a monoclonal IgG labeled with horseradish peroxidase as a detection antibody in the IgY-based immunomagnetic bead ELISA system (IgY-IMB-ELISA). The results showed that the sensitivity of IgY-IMB-ELISA was 93.3% (14 of 15) in cases of heavy infection (5000 to 9999 eggs per gram feces, *i.e*, EPG 5000–9999), 86.7% (13 of 15) in cases of moderate infection (EPG 1000–4999) and 75.0% (9 of 12) in cases of light infection (EPG <1000) of clonorchiasis. Together 36 of total 42 (85.7%) serum samples of human clonorchiasis gave a positive reaction. There was a significant correlation between ELISA optical density and egg counts (EPG) with a correlation coefficient of 0.83 in total 42 patients. There were no positive results in patients with trichinosis (n = 10) or cysticercosis (n = 10). Cross-reactivity was 6.7% (2 of 30) with schistosomiasis japonica and 10.0% (3 of 30) with paragonimiasis, respectively. No positive reaction was found in 20 healthy persons.

**Conclusions:**

Our findings suggest that IgY-IMB-ELISA appears to be a sensitive and specific assay for detection of circulating antigen in human clonorchiasis.

## Introduction

*Clonorchis sinensis*, the oriental or Chinese liver fluke, is one of the most important food-borne parasites and causes hepatobiliary diseases known as clonorchiasis. Clonorchiasis is a common food borne zoonosis with a high prevalence in Southeast Asia including China, Korea and North Vietnam [1]–[3]. Humans become infected by ingestion of metacercariae harbored in raw or under-cooked freshwater fishes. It is estimated that about 35 million people are infected with *C. sinensi*s globally among them 15 million in China [2], [4]. *C. sinensis* has been classified as one of group 1 biocarcinogens by the International Agency of Cancer Research because it is known to cause cholangiocarcinoma in humans [4]–[6]. Since clonorchiasis is one of major health problems in most endemic areas [6], [7], it is included in control programs of neglected tropical diseases (NTDs) by World Health Organization (WHO) [2].

The lack of rapid, accurate and simple-to-use for many of NTDs is an important feature for their general neglect and the under-appreciation of their disease burden [8]. Thus, accurate and reliable diagnostic tests are critical for adequate patient management and treatment. Accurate, affordable and robust diagnosis of the human liver fluke infections plays an important role in supporting surveillance of control programmes [9], [10]. Until now finding of the parasite eggs either in feces or in bile is the only diagnostic “gold standard” for clonorchiasis. The detection limit by conventional fecal examination methods was estimated to be 20 worms or approximately 1000 eggs per gram. Therefore, cases with slight infection or early infection usually are likely to be under diagnosed and conventional stool examination methods may underestimate the prevalence by as much as 20% [11], [12]. In addition, the similarity of eggs of trematodes makes the specific diagnosis difficult [13], [14]. With development of immunological diagnostic methods, detection of specific antibodies in serum samples of patients has been used widely, especially for epidemiological surveys of clonorchiasis [14], [15], though antibody detection is impossible to distinguish acute cases from past infections as the specific antibody remains for decades after the infection is cleared [16], [17].

Circulating antigens (CAg) are produced by live worms and appears earlier than antibody in blood circulation. CAg detection is a more effective way to differentiate between active and past infections [11], [18]. Several methods have been developed for detecting CAg of *C. sinensis*, including double sandwich ELISA [19], competitive inhibition ELISA [20], dot-ELISA [21], and monoclonal antibody ELISA [22]. Unfortunately, both the sensitivity and specificity of current assays for detecting of CAg are dissatisfied. *C. sinensis* adults mainly inhabit the bile ducts in the liver, compared with other flukes living in blood vessels or tissues, CAg level of *C. sinensis* in serum is lower. There is a need, therefore, to exploit and apply new knowledge and techniques in order to develop more sensitive methods for antigen detection.

Chicken egg yolk immunoglobulin (IgY) was proposed by Leslie and Clem in 1969 [23]. Compared with corresponding mammalian antibodies IgG, IgY reacts with more epitopes on a mammalian antigen and thus producing an amplification of the signal. It has been identified that IgY can improve the sensitivity of ELISA about 10–100 times [24]. IgY also has the advantage for immunodiagnostics in that it avoids the interference in immunological assays caused by the complement system, rheumatoid factors, anti-mouse IgG antibodies or human and bacterial Fc receptors [25], [26]. Based on the above advantages, IgY has been widely used to diagnosis in different diseases [26], [27].

Immunomagnetic bead ELISA is a new technology which combines ELISA with magnetic particle separation [18], [27]. Because the small size and shape of micro-beads enables them to be evenly dispersed in the liquid sample and combines with more diagnostic molecular, both immunological reaction efficiency and the sensitivity of antigen detection can be improved significantly [27], [28]. Therefore, compared with traditional ELISA plate, adsorption capacity of magnetic beads can be increased by more than 1000 times [29]–[31].

In the present study, we produced a specific polyclonal IgY from chickens immunized with recombinant cysteine proteases of *C. sinensis* (rCsCP) and coupled the specific IgY to immunomagnetic beads. Then an immunomagnetic bead sandwich ELISA based on IgY (IgY-IMB-ELISA) was developed in which IgY against rCsCP (anti-rCsCP IgY) used as a capture antibody and anti-rCsCP mouse monoclonal antibody IgG labeled horseradish peroxidase (HRP) as a detecting antibody. Finally we used IgY-IMB-ELISA to detect CAg in serum samples from patients infected with *C. sinensis* and the association between ELISA optical density (OD) and fecal egg output was examined.

## Materials and Methods

### Ethics statement

The study was approved by the Institutional Ethics Review Committees of Tongji Medical College, Huazhong University of Science and Technology, Wuhan, China (2012-IEC-S455). Written consent was obtained from all adult participants or from parents or guardians of minors. The hens were maintained in a standard SPF condition in the Experimental Animal Facility of Tongji Medical College, and the experiment was approved by the Committee on Animal Research of Tongji Medical College (SCXK2010-0009).

### Expression and purification of rCsCP

The recombinant plasmid pTrcHis cloned with cysteine proteases B of *C. sinensis* (GenBank accession: AF093242) was a kind gift of Professor Fuquan Pei from Center for Disease Control and Prevention of Guangdong Province. The recombinant plasmid was transformed into *Escherichia coli* BL21 and the recombinant proteins expressed in the bacteria were purified according to the method as described previously with minor modifications [32]. In brief, expression of rCsCp recombinant proteins was induced by adding isopropyl β-D-thiogalactopyranaside (IPTG, Sigma) at a final concentration of 0.6 mM when cultured at 37°C for 6 hours. After centrifugation, the precipitate was disrupted by sonication in 20 mM Tris-HCl buffer (pH 8.0). The recombinant protein expressed as inclusion bodies was solubilized completely with 6 M urea in 20 mM Tris-HC1 buffer (pH 8.0) and then subjected to a His Trap kit (Amersham Pharmaeia Biotech, Japan) for affinity purification according to the manufacturer’s instructions. Protein content of rCsCp was measured by BCA Protein Assay Kit (Pierce, USA). And the purified recombinant protein was analyzed by SDS-PAGE.

### Preparation and characterization of chicken anti- rCsCP IgY

The leghorn hens were immunized according to our primary scheme [33]. Briefly, leghorn hens were immunized subcutaneously with rCsCP for four times. Each hen was given 60 µg purified rCsCP in Freund's complete adjuvant (Sigma, USA) for the first immunization and three boosters of 30 µg rCsCP in Freund's incomplete adjuvant (Sigma, USA) at 2-week intervals. Eggs were collected daily before immunization and post the final immunization. Anti-rCsCP IgY was extracted from yolk according to the protocol of EGG stract IgY Purification System (Promega, USA). Protein content of anti-rCsCP IgY was measured by BCA Protein Assay Kit (Pierce, USA). The specific IgY was analyzed by SDS-PAGE and identified by Western blot, in which rCsCP was translated into nitrocellulose membrane after SDS-PAGE electrophoresis and anti-rCsCP IgY was used as the first antibody and HRP labeled anti-IgY rabbit monoclonal antibody IgG as the second antibody.

### Coupling anti-rCsCP IgY to magnetic beads

Super-paramagneticmagnetic beads (Beijing Mag-Century Biotechnology Company, China) were coupled with anti-rCsCP IgY according to our previous description [18]. In brief, the beads (500 nm in diameter) were collected from suspension media by a magnetic rack and then suspended to 0.1 M MES (2-(N-Morpholino) ethanesulfonic acid, Sigma, USA) buffer (pH 6.0) after washed twice with MES. EDC (1-ethyl-(3-dimethylaminopropyl) carboniimide hydrochloride, Sigma, USA) was added (200 µl of a 125 mg/ml solution) and the mixture was incubated with rotation for 30 min at room temperature. The beads were then washed twice with MES and resuspended to a final volume of 2.5 ml. Subsequently, the anti- rCsCP IgY (100 µl, 5.0 mg/ml) was added and the mixture was incubated with rotation for 12 h at room temperature. Finally, the magnetic beads coated with anti-rCsCP IgY were resuspended in 0.05 M PBS (pH 7.6, with 0.1% bovine serum albumin (BSA) and 0.01% sodium azide) in a final volume of 2.0 ml after washed thrice with PBS. The immunomagnetic beads (IMB) were stored at 4°C until use.

### Development of IgY-IMB-ELISA

The assay was performed at 37°C in a small flat-bottom tube fitted to a magnet rack (Bioekon Inc., Beijing, China). Each tube was added 100 µl of IMB (100 µg of magnetic beads) coated with 6.25 µg of IgY, which was used as a capture antibody, and 100 µl of serum sample was added to each tube. The tubes were incubated with rotation for 1 h and then the IMB were separated from the liquid by placing the tube on a magnetic rack. After discarding the supernatant, the IMB were washed thrice with PBST (PBS with 0.5% Tween-20). Then mAb anti-rCsCp IgG labeled HRP (Proteintech group Inc, Wuhan, China), 1: 500 diluted in PBS, was added to each tube, following with incubation for 30 min. The supernatant was discarded and the IMB were washed thrice with PBST. After the final wash, 100 µl of substrate TMB (tetramethylbenzidine dihydrochloride, Tiangen Biotech Ltd., Beijing, China) was added and incubated for 15 min. The reaction was terminated by the addition of 30 µl of sulfuric acid (2.0 M). Later, the beads were then adsorbed to the bottom of the tube by a magnet rack and the supernatant was transferred to a 96-well microtiter plate (Greine, Germany). Finally, OD values were measured at 450 nm using a MK3 microplate reader (Thermo Labsystem, Finland). Each serum sample was tested in duplicate in two repeat assays. Serum samples from healthy persons were used as negative controls and PBS as a blank control. All results were recorded after appropriate blank correction. A serum sample was considered positive if the OD value was at least two times higher than that of the negative control. The detection limit of rCsCP using IgY-IMB-ELISA was evaluated, in which rCsCP was diluted serially in PBS, ranging from 0 to 500 ng/ml and BSA used as a negative control.

### Human serum samples

A total of 42 serum samples from patients with clonorchiasis were collected for the present investigation. All cases were confirmed by stool examination with three fecal samples (Kato-Katz smears) from endemic villages for *C. sinensis* in Guangdong Province, China. Among those patients, 15 cases were defined as heavy infection (5000 to 9999 eggs per gram feces, *i.e*, EPG 5000-9999), 15 cases as moderate infection (EPG 1000-4999) and 12 cases as mild infection (EPG<1000), respectively. In addition, 80 serum samples from patients infected with other parasites were used to assess cross-reactivity, including 30 sera of human paragonimiasis from Hubei Province, 10 sera of human cysticercosis from Henan Province, 10 sera of human trichinosis from Henan Province and 30 sera of human schistoamiasis japonica from Hubei Province, China. These cases except schistosomiasis were diagnosed by patient history, clinical manifestation and serological tests. Schistosomiasis cases were confirmed as parasitologically positive by using the Kato-Katz method with three fecal samples or by miracidial hatching assay. Serum samples of 20 healthy persons living in Shandong Province (non-endemic for clonorchiasis) were obtained and used as controls. All sera were stored at −20°C until use.

### Data analysis

The sensitivity of our assay was defined as the percentage of serum samples among human clonorchiasis showing an OD value two times higher than that of the negative control. The rate of cross-reactivity was defined as the number of persons who had positive results as a proportion of the total number of persons without clonorchiasis but with other common helminth infections, which were represented by persons with paragonimiasis, cysticercosis, trichinosis and schistoamiasis in the present study. OD value data were expressed as means±SDs and analyzed with SPSS 13.0. Comparison among different groups was made with ANOVA. *P* values <0.05 were considered significant and *P* values <0.01 as highly significant.

## Results

### Expression and purification of rCsCP

**Figure 1:**
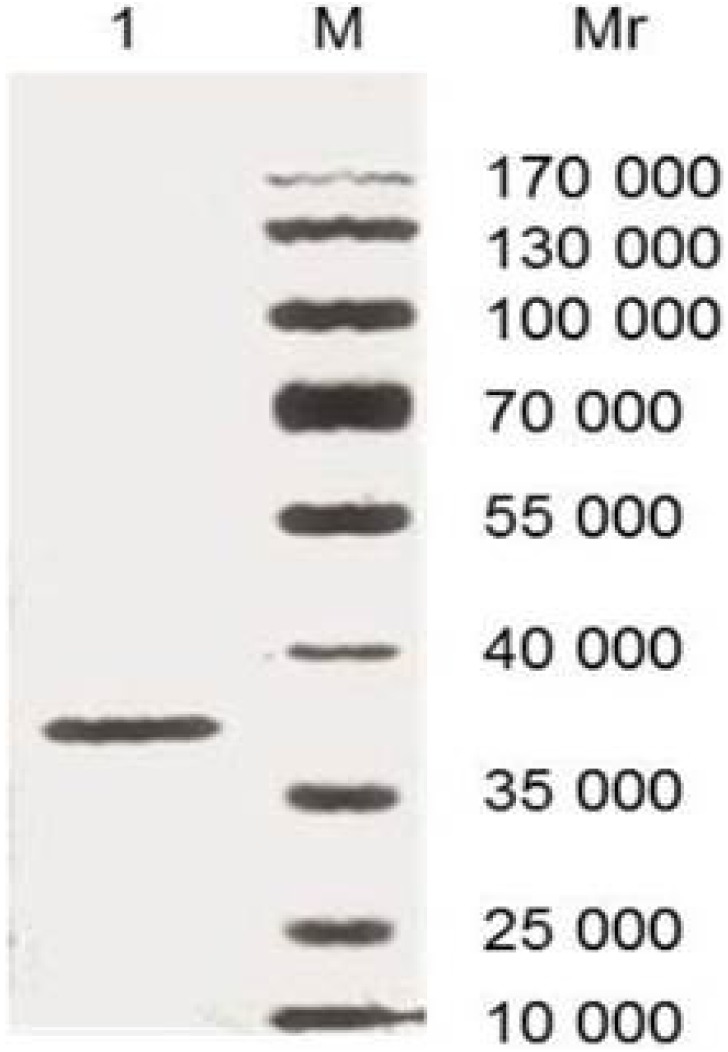
Analysis of rCsCP by 12% SDS-PAGE in non-reducing condition. 3 µg of protein was loaded for each lane. M: Protein molecular weight standards marker; 1: Purified rCsCP.

As Figure 1 shown, SDS-PAGE analysis showed recombinant proteins with a 37 KDa band, which indicated that purified rCsCp was produced successfully.

### Purification and characterization of anti-rCsCp IgY

**Figure 2:**
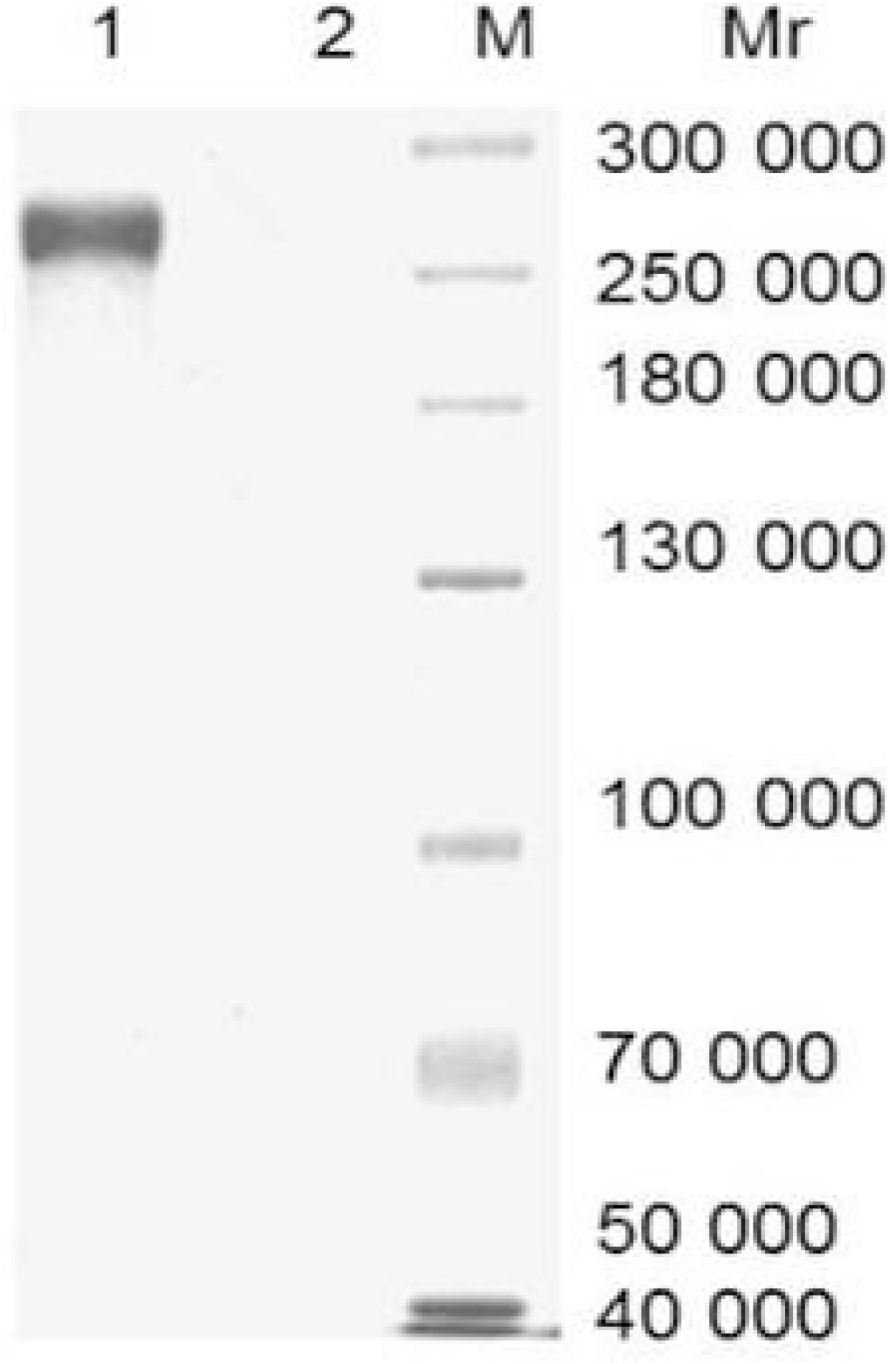
Analysis of IgY by 12% SDS-PAGE in non-reducing condition. 4 µg of protein was loaded for each lane. M: Protein molecular weight standards marker; 1: IgY from immunized egg yolk; 2: IgY from non-immunized egg yolk.

**Figure 3:**
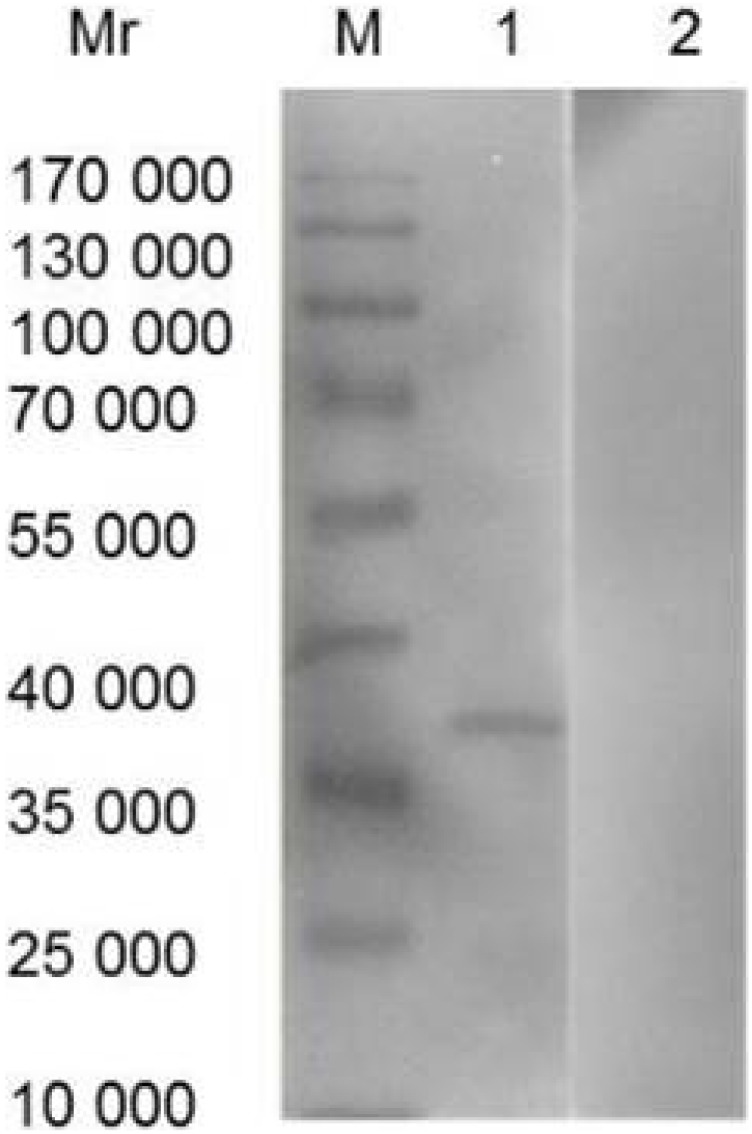
Analysis of IgY by Western blot. M: Protein molecular weight standards marker; 1: rCsCP probed with purified IgY from immunized egg yolk; 2: rCsCP probed with purified IgY from non-immunized egg yolk.

On average, 60 mg of IgY was extracted from each immunized egg yolk. IgY concentration varied from 3.8 to 6.9 mg/ml of egg yolk. A non-reduced SDS-PAGE analysis showed a protein band with molecular weight of 270 kDa characteristic of the specific IgY while no band for non-immunized chicken egg yolk (Figure 2). The analysis of Western blot showed that IgY from immunized egg yolk recognized rCsCp specifically which was indicated by an immunoreative band at 37 kDa, while no reaction between non-immunized IgY and rCsCp (Figure 3).

### IgY-IMB-ELISA

**Figure 4:**
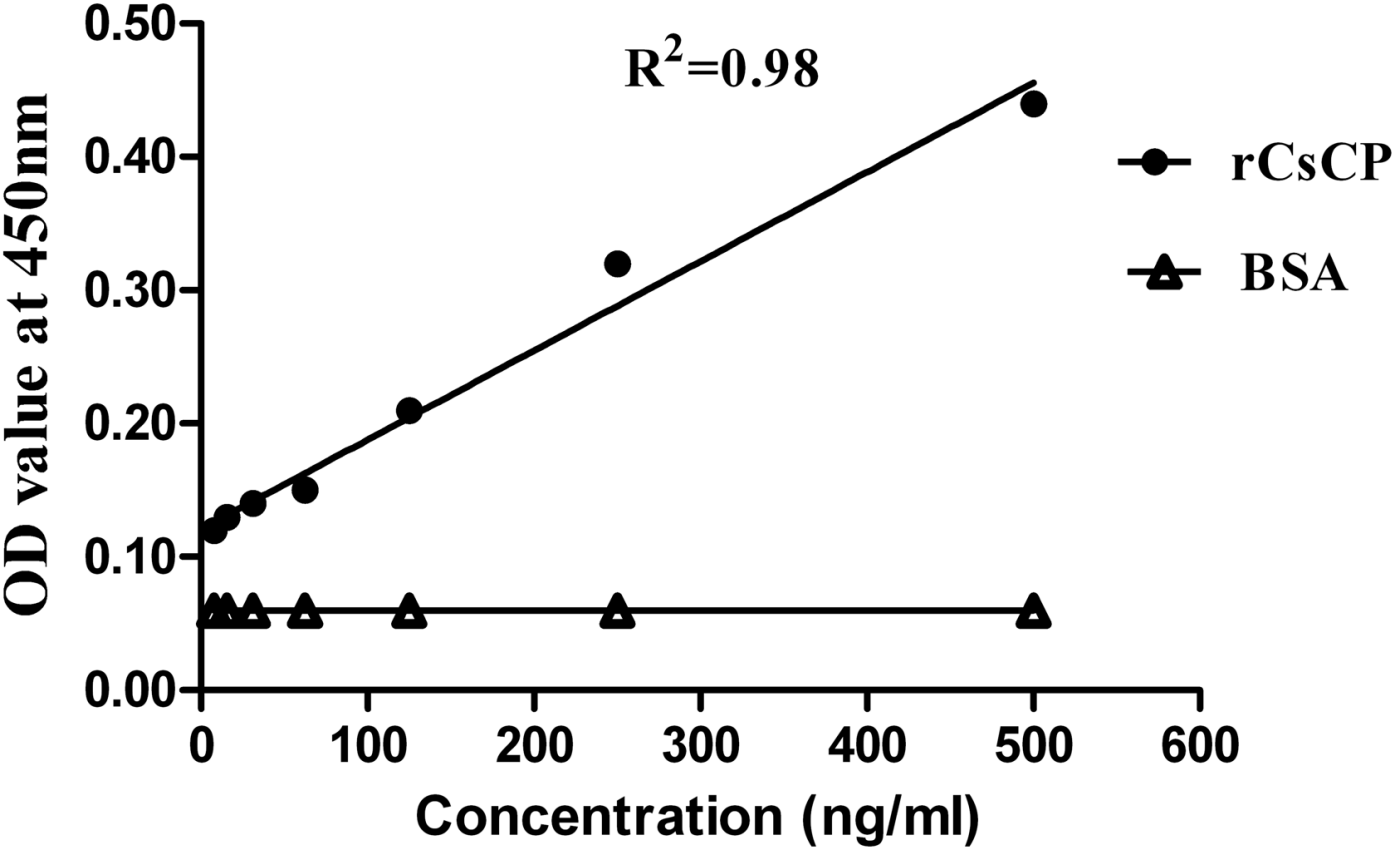
Relationship between rCsCP concentrations and OD values. Bovine serum slbumin (BSA) was used as a negative control. OD values of rCsCp are expressed as mean OD values measured with IgY-IMB-ELISA from two independent combined experiments.

To establish the detection limit and linearity of IgY-IMB-ELISA, a serially diluted rCsCp ranging from 0 to 500 ng/ml were used. As shown by the standard curves (Figure 4), the detectable limit for rCsCp using the assay was 7.8 ng/ml and OD values of samples were positive correlated with concentrations of rCsCp (The correlation coefficient 0.98). Furthermore, the results indicated that IgY was specifically bound to rCsCp.

**Figure 5:**
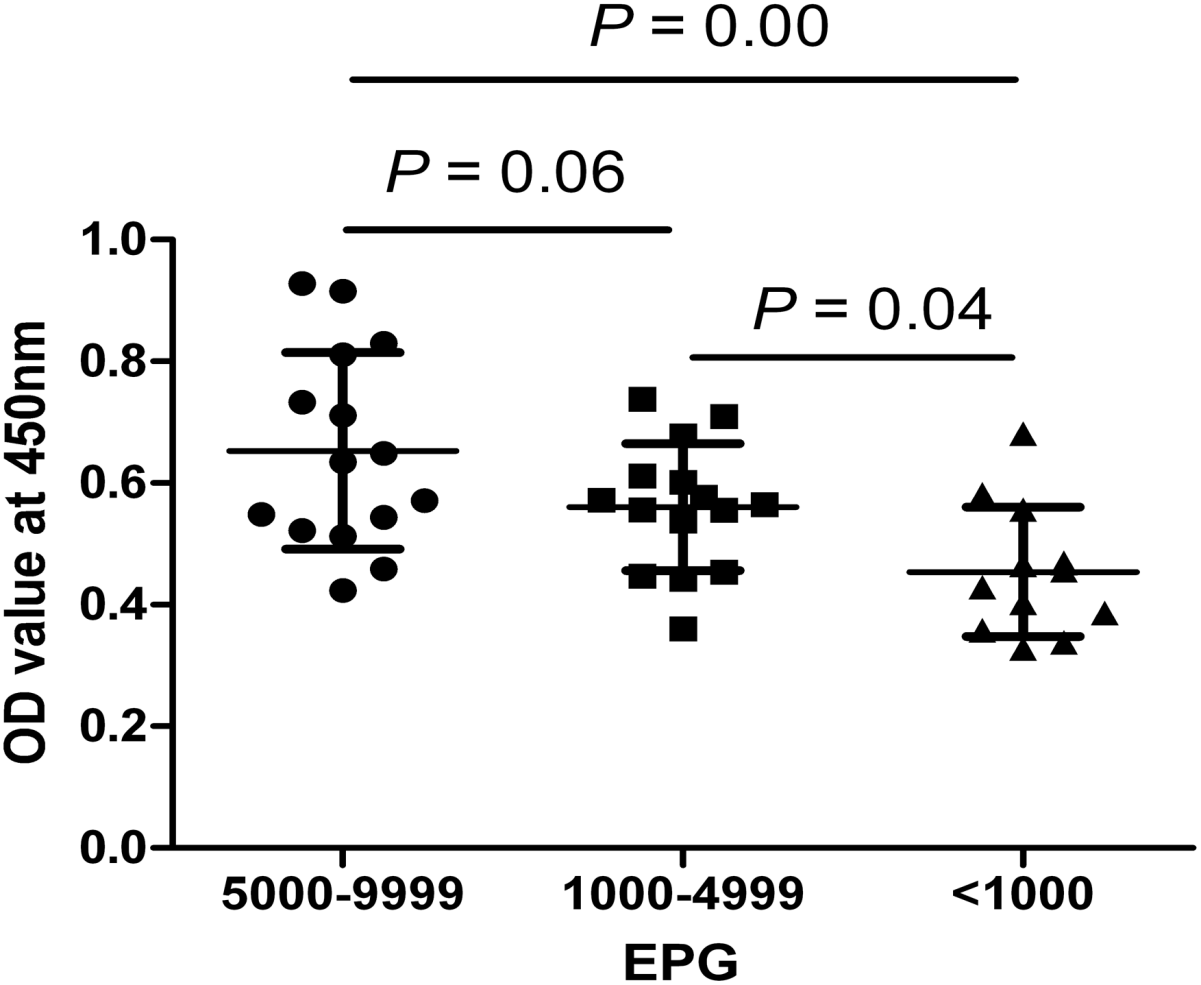
Comparison of OD values obtained in IgY-IMB-ELISA for detection of serum circulating antigens from clonorchiasis patients with different infection intensity (egg per gram of feces, EPG).

**Figure 6:**
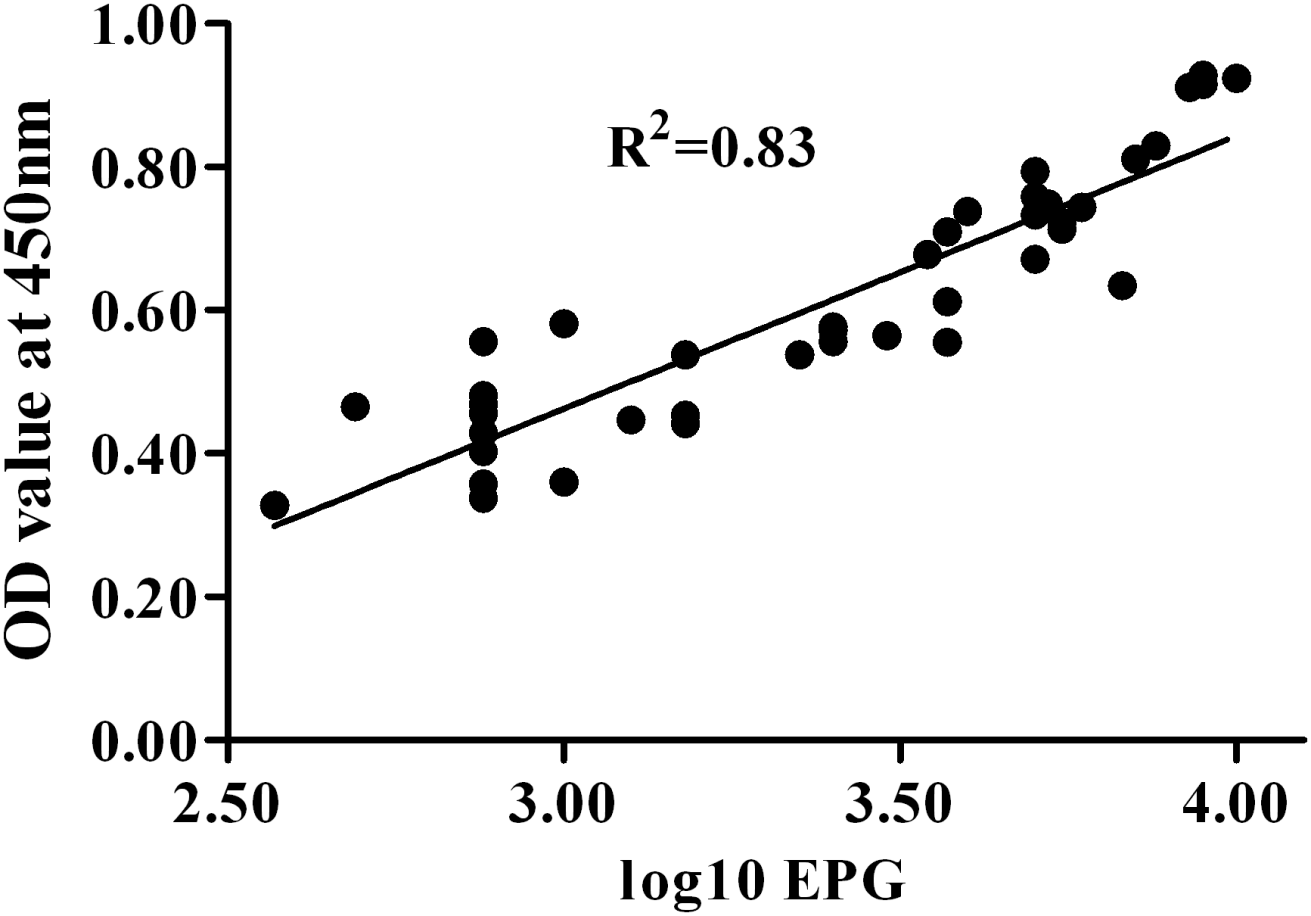
Correlation between OD values obtained in IgY-IMB-ELISA and *Clonorchisis sinensis* egg counts (egg per gram of feces, EPG) from human clonorchiasis. The EPG ranged from 375 to 9500, which is represented by their logarithmic transformation along the x-axis. A high correlation was found between the OD values and egg counts (R^2^ = 0.83).

A total of 42 sera of human clonorchiasis were tested using IgY-IMB-ELISA to detect the circulating antigens. The results (Table 1) showed that the sensitivity of IgY-IMB-ELISA was 93.3% (14 of 15) in cases of heavy infection (EPG 5000-9999), 86.7% (13 of 15) in cases of moderate infection (EPG 1000-4999) and 75.0% (9 of 12) in cases of light infection (EPG <1000) of clonorchiasis. Summarizing these above data, the sensitivity of IgY-IMB-ELISA for detection of human clonorchiasis was 85.7% (Table 2). As Figure 5 shown, the difference between OD values of heavy infection (EPG 5000-9999) and those of light infection (EPG <1000) was highly significant. OD values of moderate infection (EPG 1000-4999) were significantly higher than those of light infection. However, the difference between OD values of heavy infection and those of moderate infection was not statistically significant although heavy infection had higher values than moderate case. Furthermore, there was a significant correlation between ELISA OD values and egg counts (EPG) and a correlation coefficient of 0.83 in total 42 patients (Figure 6).

**Table 1:** Detection results in sera of human clonorchiasis with different egg per gram faces (EPG) by IgY-IMB-ELISA.

Human clonorchiasis		Positive	
EPG	No.	No.	Percentage (%)
<1000	12	9	75.0
1000–4999	15	13	86.7
5000–9999	15	14	93.3

Each sample had duplicate tubes and the experiment was repeated twice.

**Table 2:** Detection results in sera from patients infected with different helminthes by IgY-IMB-ELISA.

Detected groups		Positive	
Patients	No.	No.	Percentage (%)
Clonorchiasis	42	40	85.7
Trichinosis	10	0	0
Cysticercosis	10	0	0
Schistosomiasis	30	2	6.7
Paragonimiasis	30	3	10.0
Healthy persons	20	0	0

Each sample had duplicate tubes and the experiment was repeated twice.

There was no positive results in patients both of trichinosis (n = 10) and of cysticercosis (n = 10). Cross-reactivity was 6.7% (2 of 30) with schistosomiasis japonica and 10.0% (3 of 30) with paragonimiasis. Together, the specificity of the novel assay based on IgY was 93.3% (75 of 80). In addition, no positive reaction was found in 20 healthy persons (Table 2).

## Discussion

Early diagnosis and treatment are essential to prevent serious complications of clonorchiasis in humans [34]. Detection of antigens secreted from the parasite is in principle an interesting diagnostic alternative for diagnosis of parasitic diseases since the antigens are secreted by living parasites, thus it is a more effective way to differentiate between active and past infections and to evaluate the efficacy of chemotherapy. Several assays have been developed for detection of circulating *C. sinensis* adult worm antigens in sera of animals and humans but the sensitivity of those assays was lower, the minimum detectable amount of the antigens ranged from 0.03 µg/ml to 12.5 µg/ml [19]–[22]. The currently available diagnostic methods for liver fluke detection are still far from ideal and significant problems are seen in areas with low prevalence, light infections [17]. Therefore, it is still a challenge to explore a method with better sensitivity and specificity to diagnose cases of clonorchiasis especially lightly infected ones.

Cysteine protease of *C. Sinensis* (CsCp) has been proved to distribute surrounded the intestine of adult worm, excretory bladder and tegument of metacercaria, as well as oral sucker, excretory bladder and tegument of cercaria [35]. CsCp has been proved to play key roles in nutrient uptake, excystment/encystment, host tissue invasion, larval migration, and immune evasion [36]. CsCp is mainly located in the intestine of adult worm and it is the main ingredient of *C. sinensis* excretory-secretory products. *C. sinensis,* as a fluke without an anus, has to regurgitate to eliminate its residual products of intestines and such gut associated molecules are more expected to enter the circulation of the host compared with the crude adult worm antigens [35]. Therefore, CsCp has been used as a candidate antigen for serodiagnosis or candidates for vaccine [36]–[38]. In this study, immunization of hens with rCsCp of *C. sinensis* presented the specific IgY with an average amount of 60 mg from each egg yolk. The analysis of the recovered IgY by SDS-PAGE showed a good purity, which indicates that leghorn hens can be the ideal host for the production of anti-rCsCp IgY. The analysis of Western blot showed that the anti-rCsCp IgY could recognize CsCp specifically. Together the results show hens have the remarkable ability to rapidly and efficiently generate an abundant IgY and provide specific IgY in a noninvasive way.

Chicken IgY reacts with more epitopes on a mammalian antigen and therefore gives an amplification of the signal because of the evolutionary difference between mammals and birds [24], [26]. In addition, immunomagnetic beads of high quality improve the effectiveness of antibody conjugation, thereby enhancing the sensitive capacity of the traditional ELISA [30]. Both ideas have been confirmed by our previous study in the diagnosis of schistosomiasis [18], [27]. Those research works make us to further utilize advantages of IgY and magnetic beads for development of an more efficient diagnostic reagent for clonorchiasis. In the experiment, we prepared a specific anti-rCsCP IgY and on the basis further developed an novel IgY-IMB-ELISA which combined advantages of IgY and immunomagnetic beads to detect the circulating antigens of *C. sinensis*. The result showed that minimum detectable amount of antigen (rCsCP) was 7.8 ng/ml, which suggests that the detectable levels of circulating antigens could be increased from previous µg/ml to ng/ml level in human serum. Mazidur et al [34] reported that the detection limit of a coproantigen capture ELISA was 20 ng/ml in sample buffer and 40 ng/ml in uninfected fecal extract. That assay could detect antigens of *C. sinensis* in feces of rats that were infected with a single worm. Together those data indicate that our ELISA system is highly sensitive in detecting antigens and may eventually be used to diagnose low-intensity infection, due to the advantages of both IgY and immunomagnetic beads. Furthermore, this assay can be used to evaluate the circulating antigens existence and level because the OD values increase linearly with both rCsCP concentrations and EPG. It’s quite well known that EPG correlates with the worm burden in human clonorchiasis [39]. Assessment of infection intensity is critical to understanding density-dependent regulatory mechanisms of parasite transmission and morbidity, which plays a center role in determinants of transmission dynamics, morbidity, and disease burden [8], [10]. Therefore, there is another advantage of the IgY-IMB-ELISA in that it can detect active infections, as it demonstrates the presence of the parasite directly.

We used IgY-IMB-ELISA to diagnose *C. sinensis* infection in serum samples of patients and the results showed a sensitivity of 85.7% and a specificity of 93.3%. As for the cross-reaction with schistosomiasis and paragonimiasis in our study, it may arise from the common conformational epitopes in cysteine protease of *C. sinensis* and other parasites but they can be distinguished easily from clonorchiasis according to their different medical history, clinical signs and symptoms. Although both other important liver fluke infections fascioliasis and opisthorchiasis are supposed to be high cross-reactivity to human clonorchiasis in immunological diagnosis, each species has a particular geographical distribution which is different from clonorchiasis endemic areas [40], [41]. *Opisthorchis viverrini* is mainly endemic in Thailand, Laos, Cambodia and Central Vietnam [40] while *Fasciola hepatica* is a major concern in the Americas, Europe and Oceania [41].

Only a few antigen-based detection systems were explored for *C. sinensis* diagnosis of human clonorchiasis [19]–[22]. The sensitivities of those methods varied depending on methods used, antigens targeted, and infection intensities of population examined. A recent study for *C. sinensis* in an animal model suggested that coproantigen detection is a promising approach for future diagnosis of light infection as well as post chemotherapy evaluation [34]. The present study showed a higher sensitivity (7.8 ng/ml versus 20 ng/ml) in the antigen detection limit. Well-established molecular techniques such as polymerase chain reaction (PCR) tests were shown by different laboratories to be sensitive for lower-intensity infections [42], [43]. However, the requirement of expensive equipment and professionally trained technicians may impair their application for mass surveillance in many rural areas according to the ASSURED (affordable, sensitive, specific, user-friendly, robust and rapid, equipment-free and deliverable) characteristics for a diagnostic test recommended by WHO [8]. Our IgY-IMB-ELISA based on antigen detection shows advantages of its improved sensitivity, compliance, and practicability for both patients and technicians. These benefits and improvements indicate that the IgY-IMB assay is an easy-to-handle, specific and stable approach in diagnosis of clonorchiasis.

Taken together, to our best of knowledge, this is the first time to develop an antigen-based detection assay based on IgY and immunomagnetic beads for diagnosis of human clonorchiasis. Our results show that IgY-IMB-ELISA is sensitive and specific for the detection of circulating antigen in patients infected with *C. Sinensis* and might be a strong supplement or replacement for laborious parasitologic methods in diagnosis of human clonorchiasis, especially in the application of field surveillance. Further studies will be focused on the precision and stability of the IgY-IMB-ELISA and evaluation of the assay by assessment of chemotherapy efficacy in human clonorchiasis.
